# Buffalo Colleges

**Published:** 1886-10

**Authors:** 


### *BUFFALO COLLEGES.*

The Buffalo colleges have, all during the present month, opened their doors to the students and, as usual the old schools offer increased facilities. Both medical colleges have added new instructors or professorships to their courses. The Buffalo Medical College opened on Sept. 23d and will continue till Feb. 23d. There is the usual class in attendance, numbering from 120 to 130. At the opening exercises Dr. Hinkel delivered an address and called especial attention to the advances made in laryngology. He announced that Dr. F. P. Vandenburgh, who graduated from the college two years previous and who had been an assistant to the Professor of Chemistry, would succeed Prof. Withaus as Professor of Chemistry; this, however, was a mistake and should have been that Dr. Vandenburgh would lecture on elementary chemistry and Prof. Withaus would lecture on medical chemistry. It was understood that Prof. Withaus would necessarily be absent from the city more on account of his having been appointed Prof. of Chemistry in the New York University.

The College of Pharmacy was opened the night previous, and several interesting addresses were delivered. Dr. Vandenburgh, the secretary, delivered the address on the part of the faculty. He called attention to the fact that women would be admitted to the college and that two had entered this course. He spoke of the especial qualifications of women for this profession, and thought they were even better adapted for this than for the medical branch. This, however, is very questionable. There must be in the minds of every one a serious doubt as to the advisability of women becoming drug clerks. The long hours and constant standing being very harmful. Yet, we are all glad of this opportunity for them to try to demonstrate which be true.

The Niagara Medical College opening occurred on Sept. 29th There was a very large attendance of students and their friends. They were well entertained and instructed by Prof. Stockton. There is every prospect of a very large class. The course will continue till April 15th. Want of space prevents our giving an extended notice in this number.

